# Evaluation of Glycerol Concentration in the Production of Lemon Oil Incorporated Pectin-Based Films Using Principal Component Analysis

**DOI:** 10.3390/foods14091576

**Published:** 2025-04-30

**Authors:** Belkis Akachat, Louiza Himed, Assala Torche, Yahia Khelef, Malika Barkat, Merniz Salah, Maria D’Elia, Luca Rastrelli, Pınar Terzioğlu

**Affiliations:** 1 Laboratory of Biotechnology and Food Quality (BIOQUAL), Department of Food Biotechnology, Institute of Nutrition, Food and Agro-Food Technologies (INATAA), Freres Mentouri University 1, Constantine 25000, Algeria; belkis.akachat@doc.umc.edu.dz (B.A.); barkat.inataa@yahoo.com (M.B.); 2 Research Laboratory “Protection of Ecosystems in Arid and Semi-Arid Zones ECOSYS”, KASDI University Merbah, Ouargla 30000, Algeria; labo-ecosys@univ-ouargla.dz; 3 Laboratory Biology, Environment and Health (LBEH), Department of Cellular and Molecular Biology, Faculty of Natural Science and Life, University of El Oued, El Oued 39000, Algeria; khelef-yahia@univ-eloued.dz; 4 Food Sciences Laboratory, Formulation Innovation Valorization and Artificial Intelligence (SAFIVIA), Constantine 25000, Algeria; 5 Institute of Industrial Hygiene and Safety, University Batna 2, Batna 05078, Algeria; s.merniz@univ-batna2.dz; 6 National Biodiversity Future Center (NBFC), 90133 Palermo, Italy; mdelia@unisa.it; 7 Department of Pharmacy, University of Salerno, Via Giovanni Paolo II, 132, Fisciano, 84084 Salerno, Italy; 8 Dipartimento di Scienze della Terra e del Mare, University of Palermo, 90123 Palermo, Italy; 9 Department of Polymer Materials Engineering, Faculty of Engineering and Natural Sciences, Bursa Technical University, 16310 Bursa, Türkiye; pinar.terzioglu@btu.edu.tr

**Keywords:** biopolymer-based packaging, glycerol, pectin, plasticizer concentration, physical properties, lemon oil

## Abstract

This research explores how varying glycerol concentrations (0–30 wt%) affect the physicochemical and mechanical characteristics of pectin films, derived from *Citrus limon* waste and enriched with lemon essential oil. The films were produced using the casting method. The findings show that glycerol significantly impacts film thickness, swelling behavior, water solubility, moisture content, water vapor permeability, and structural and mechanical characteristics. FTIR spectroscopy confirmed molecular interactions between glycerol and the film matrix. Notably, films with 20–30 wt% glycerol had reduced transparency. Mechanically, glycerol increased the elongation at break, enhancing flexibility, while a 5 wt% glycerol concentration optimized tensile strength. However, higher glycerol levels led to decreased tensile strength. Principal Component Analysis identified 5 wt% glycerol as optimal for balancing flexibility and structural integrity. Additionally, glycerol-plasticized films were more hydrophilic than the control. These results highlight glycerol’s crucial role as a plasticizer and the importance of precise concentration control in biodegradable film formulations.

## 1. Introduction

The increasing environmental concerns surrounding plastic waste have underscored the urgent need for biodegradable alternatives. Biodegradable packaging plays a crucial role in mitigating plastic pollution, as it decomposes naturally, thereby reducing landfill accumulation and minimizing environmental impact. Biopolymer-based biodegradable materials not only decrease the volume of non-degradable waste but also contribute to soil enrichment through composting [1,2]. Additionally, advancements in biodegradable packaging technologies are driving the development of sustainable solutions that support a circular economy, promoting the utilization of renewable resources while improving food preservation [3]. The widespread adoption of biodegradable packaging represents a fundamental step toward achieving a more sustainable future. Pectin, a natural polysaccharide found in the cell walls of various fruits, has attracted growing interest in sustainable food packaging applications due to its biodegradability [4]. Pectin is particularly valued for its film-forming capability, biocompatibility, and non-toxic nature, making it an excellent candidate for eco-friendly packaging solutions [5,6,7]. Recent studies have shown that pectin-based films can be efficiently produced from agricultural by-products, including citrus peels and apple pomace, which are considered primary sources for industrial pectin extraction due to their high yields. In addition to these conventional sources, alternative non-traditional raw materials have been explored, including pineapple peels, eggplant peels, pomegranate peels, cocoa pod husks, banana peels, and tomato husks. Furthermore, tropical fruits, such as mango peels, passion fruit rinds, and jackfruit peels, have been identified as promising sources for pectin extraction [6,7,8]. The diversification of pectin sources not only enhances its production but also contributes to food industry waste reduction, aligning with sustainable waste valorization strategies. The extraction of pectin from these by-products adds value to agricultural residues while supporting the development of biodegradable materials for various applications.

Encapsulating lemon essential oil within a pectin matrix offers multiple advantages, enhancing both the functionality and stability of the oil in biodegradable films. Pectin provides an effective encapsulation medium due to its biocompatibility and gel-forming properties, which protect the volatile compounds of lemon essential oil from degradation [9]. Additionally, the encapsulation process enables a controlled release mechanism, allowing the antimicrobial properties of lemon essential oil to be gradually released over time, thereby extending the shelf life of food products [10]. Our previous study demonstrated that the incorporation of lemon essential oil into pectin-based films significantly influenced their physicochemical and functional properties, improving flexibility, water resistance, and antimicrobial activity against foodborne pathogens and spoilage microorganisms. Furthermore, these films exhibited increased opacity, moderate antibacterial activity, and strong antifungal effects, highlighting their potential as active food packaging materials [11].

Pectin has gained considerable attention as a promising biopolymer for food packaging, owing to its distinct characteristics, including biodegradability, biocompatibility, and film-forming ability. Furthermore, pectin-based packaging materials are generally regarded as safe and are classified as Generally Recognized as Safe (GRAS) by the U.S. Food and Drug Administration (FDA) [12,13].

However, the inherent brittleness and hydrophilic nature of pectin films may negatively impact their mechanical and barrier properties, limiting their suitability for applications in food packaging. To address these challenges, plasticizers such as glycerol, polyethylene glycol, propylene glycol, and sorbitol are often incorporated to improve the flexibility and processability of the films, thereby enhancing their overall performance and broadening their applicability in food packaging [14].

Among these plasticizers, glycerol has been widely recognized as a key additive in the formulation of biodegradable films due to its low molecular weight, polarity, and water solubility, making it particularly suitable for use with water-soluble polymers [15]. Glycerol acts by increasing the mobility of polymeric chains, reducing intermolecular forces, and thereby enhancing film flexibility. However, this plasticizing effect is often accompanied by a decrease in tensile strength, stiffness, and gas barrier properties [16]. Differential scanning calorimetry (DSC) analyses have shown that glycerol enhances the mobility and free volume within polymeric matrices, leading to an increase in moisture content and film thickness. Additionally, previous studies have demonstrated a negative linear correlation between glycerol concentration and both tensile strength and Young’s modulus, indicating that higher glycerol levels contribute to reduced mechanical strength [17].

It is well established that plasticizer concentration plays a key role in determining the physicochemical characteristics of biodegradable packaging materials. However, to date, no comprehensive study has systematically evaluated the effect of different glycerol concentrations on the structural, mechanical, and barrier properties of pectin-based films. This study aims to investigate the impact of varying glycerol concentrations (0, 5, 10, 20, and 30 wt%) on pectin films enriched with encapsulated lemon essential oil. The findings of this research may provide new insights into optimizing the formulation of pectin-based films for applications in food packaging.

## 2. Materials and Methods

### 2.1. Materials

Pectin was extracted from essential oil extraction waste using a hot acid solution with hydrochloric acid (HCl). Glycerol and citric acid were purchased from Isochem, Angamaly (India).

### 2.2. Preparation of Encapsulated Lemon Essential Oil (LEO)

Lemon essential oil was extracted from lemon peel waste using a Clevenger hydro-distillation apparatus (Jeulin, France). The essential oil was encapsulated within a pectin matrix to enhance its stability and functionality. To achieve this, pectin was dissolved in an aqueous solution containing citric acid (0.5% *w*/*v*) to obtain a pectin solution (5% *w*/*v*). Subsequently, lemon essential oil (1% *v*/*v*) was incorporated into this solution. The essential oil droplets were uniformly dispersed within the pectin matrix by homogenizing the mixture with a magnetic stirrer at 50 °C for half an hour at 4500 rpm. Following emulsification, the emulsion was frozen at −70 °C overnight. The stabilized emulsion was lyophilized by a freeze-drying system at −55 °C under a vacuum pressure of 0.15 mm Hg for 2 days, effectively removing water content and yielding powdered stable microcapsules [18].

### 2.3. Preparation of Pectin Films with Varying Glycerol Concentrations

**Figure 1:**
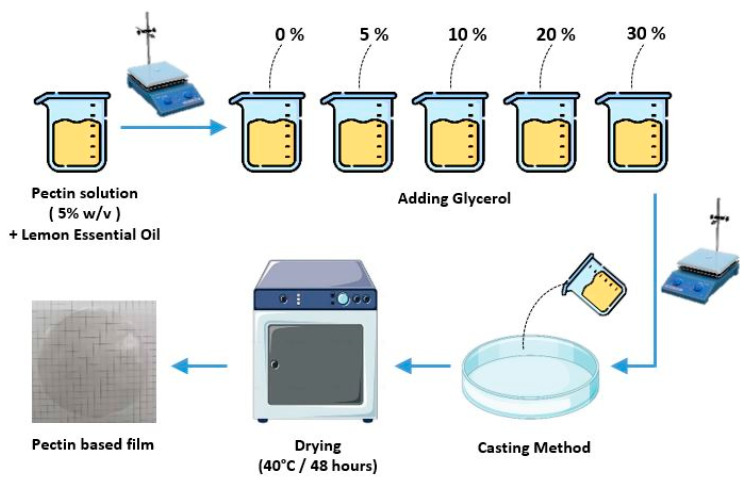
Schematic illustration of active pectin film preparation by solvent casting method.

The solvent casting method was used to develop pectin-based films according to Akachat et al. [18], as illustrated in Figure 1. This process involved several systematic steps to ensure optimal film formation. First, a 5% (*w*/*v*) pectin powder solution was prepared by dissolving pectin in distilled water, with 10 wt% citric acid (relative to the total mass of water) and 1% (*w*/*v*) encapsulated essential oil. The solution was heated to 65 °C and continuously stirred using a magnetic stirrer for 30 min to achieve complete homogenization.

Subsequently, different concentrations of glycerol (0–30 wt% relative to the total pectin mass, as shown in Table 1) were added to the pectin solution. The final film-forming solution was mixed for an additional 30 min under the same heating conditions to ensure thorough integration of glycerol into the pectin matrix.

A 20 mL aliquot of the film-forming solution was then cast onto round PET Petri dishes (90 × 90 mm). The dishes were placed in an oven set at 40 °C for 2 days to allow controlled drying and solidification of the films. Once dried, the films were carefully peeled from the Petri dishes and stored for further characterization.

The pectin-based films were labeled according to increasing glycerol content as PC, PGLY1, PGLY2, PGLY3, and PGLY4, as detailed in Table 1.

### 2.4. Film Thickness

**Figure 2:**
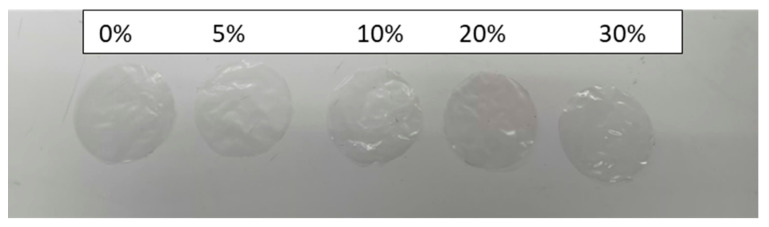
Pectin films with varying glycerol concentrations.

The thickness of the films (Figure 2) was determined using a handheld digital micrometer (ABS ASIMETO, Istanbul, Türkiye). Ten measurements per sample were taken at randomly selected points. The average value was calculated and recorded to ensure precision.

### 2.5. Moisture Content

In order to determine the moisture content of the films, square film samples (5.0 cm × 5.0 cm) were dried in an oven at 105 °C until a constant weight was reached [15]. The moisture content was calculated using the equation provided in Equation (1):

(1) $$Moisture\;content=\frac{Mw-Md}{Mw}\times 100\%$$

Here, *Mw* refers to the film weight stabilized at 75% relative humidity (RH), while *Md* denotes the film dry weight.

### 2.6. Solubility

Film solubility was determined using a standardized immersion method. Each film sample was cut into uniform pieces (2 × 2 cm). Prior to testing, the films were pre-dried at 60 °C until a constant weight was reached in order to minimize the influence of residual moisture on the solubility measurement.

The dried films were then immersed in distilled water and kept at room temperature for a specified duration. After immersion, the films were carefully removed, blotted with filter paper to eliminate surface water, and again dried in an oven at 60 °C until they reached a constant weight.

The solubility percentage was calculated based on the weight loss before and after immersion using the following Equation (2):

(2) $$S=\frac{I_{w}-I_{f}}{I_{w}}\times 100$$

where $I_{w}$ is the initial dry weight of the film and $I_{f}$ is the final dry weight after immersion and re-drying.

It is important to note that the weight loss measured may result from both actual film dissolution and the loss of water that was initially absorbed in the film matrix. Therefore, while this method provides a useful estimation of solubility, it may include a combined effect of these factors. Five measurements were taken for each sample to ensure accuracy and reproducibility.

### 2.7. Swelling Ratio

The swelling ratios of the film were determined using the method described by ASTM D570 [19]. Film samples, weighed beforehand, were immersed in water at 25 °C for two minutes. The films were reweighed after gently blotting off any excess surface water with filter paper. The swelling ratio was calculated by comparing the weight gain to the initial weight of the film. This method is widely used in polymer film studies to assess how water absorption affects material properties. The swelling ratio was calculated using the equation provided in Equation (3):

(3) $$\mathrm{Swelling}\;\mathrm{Ratio}\;(\%)=\frac{w_{i}-w_{f}}{W_{f}}\times 100$$

where $w_{f}$ is the weight of the film after immersion in water (g) and $w_{i}$ is the initial weight of the film before immersion (g).

### 2.8. Water Vapor Permeability

Water vapor permeability (WVP) was measured gravimetrically following the method described by Ilyas et al. [20]. Circular film samples were sealed over the openings of centrifuge tubes containing 5 g of desiccant-grade silica gel. These tubes were then placed inside desiccators containing 200 mL of deionized water to maintain a constant relative humidity of 100% at a controlled temperature of 20 °C (Figure 2). The weight of each sample was recorded daily over seven consecutive days. Each WVP measurement was performed in triplicate to ensure accuracy, and the results were expressed in g m^−1^ s^−1^ Pa^−1^, using Equation (4):

(4) $$\mathrm{WVP}\frac{W\times X}{A\cdot \Delta P\cdot t}$$

In this equation, *W* represents the weight increase of the centrifuge tube (g), *X* is the thickness of film (m), *t* is the duration of the test (s), *A* is the permeation area of the film (m^2^), and Δ*P* denotes the difference in water vapor pressure across the film sample (2339 Pa).

### 2.9. Contact Angle Measurements

The water contact angle of the films was studied by an Attension Theta Lite optical tensiometer (Biolin Scientific, Gothenburg, Sweden) equipped with an automatic dispenser. A 5 μL drop of distilled water was carefully placed on the surface of 2 × 2 cm film samples, following the method described by Remedio et al. [21]. All measurements were conducted in triplicate, and the reported values reflect the average of three separate readings.

### 2.10. Film Transparency

The light barrier properties of pectin films were assessed for both visible and ultraviolet (UV) radiation. A rectangular film strip (9 mm × 40 mm) was placed in a Cary Series UV-VIS Spectrophotometer, and transmittance was measured at a wavelength of 600 nm. Film transparency was then calculated using the following Equation (5):

(5) $$T=\frac{log(\%T600)}{L}$$

where %*T*600 = the percent transmittance, and *L* is the thickness of the sample (mm).

### 2.11. Film Transmittance

The transmittance spectra of the films were recorded over the 200–800 nm wavelength range with a 1 nm step size using a Cary 100/G9821A UV–VIS spectrophotometer (Agilent Technologies, Santa Clara, CA, USA).

### 2.12. Fourier Transform Infrared Spectroscopy (FT-IR)

Fourier Transform Infrared Spectroscopy (FT-IR) was performed to identify interactions as well as specific functional groups within the films. The spectra were recorded using a Thermo Nicolet iS50 FT-IR spectrometer, Waltham, Massachusetts, USA scanning within the wavenumber range of 650–4000 cm^−1^.

### 2.13. Mechanical Properties

The mechanical properties of the pectin films were assessed to evaluate the effect of glycerol incorporation. Tensile strength, elongation at break, and Young’s modulus (YM) were measured with the help of a universal testing machine (AGS-X Series, Shimadzu, Kyoto, Japan), following the ASTM D882 standards [22]. The tests were conducted with a crosshead speed of 10 mm/min and a 1 kN load cell. Each measurement was repeated five times, and the results were reported as mean values with standard deviations [23]. To ensure the reliability and reproducibility of tensile strength measurements, all film samples were prepared, conditioned, and tested under strictly controlled environmental conditions. Specifically, all films were cast from the same batch of film-forming solution and dried in a climate-controlled chamber maintained at approximately 25 °C and 50% relative humidity. Prior to mechanical testing, the samples were equilibrated under the same conditions for at least 48 h. Tensile tests for each group were conducted on the same day, using the same universal testing machine and calibration settings, to minimize variability due to instrumental or environmental changes. This procedure was adopted to account for the high sensitivity of bio-based materials to ambient factors such as temperature and humidity.

### 2.14. Burst Strength

The burst strength of films was assessed using a burst strength tester TA-HD Plus Texture Analyzer (Stable Micro Systems Co., Ltd., Godalming, UK). Film samples were prepared by cutting them into rectangular shapes measuring 2 cm × 2 cm. These samples were then secured onto a film support ring with a diameter of 10 mm, which was mounted on a heavy-duty platform (HDP/FSR). The bursting test was conducted using a ball probe (SMS P/0.25S) with a diameter of 6.25 mm. To ensure accurate alignment, the film support rig was positioned to allow the ball probe to move centrally through the aperture. The testing commenced with the probe moving at a speed of 0.2 mm/s. During the test, the maximum force required to rupture each film sample was recorded, along with the maximum distance the probe traveled before rupture occurred [24].

### 2.15. Film Morphology

The surface morphology of the films was examined using a field emission scanning electron microscope (FE-SEM; VEGA 3, TESCAN, Orsay, France). Prior to imaging, the samples were sputter-coated with a thin layer of gold to enhance conductivity. SEM imaging was performed at an accelerating voltage of 20.0 kV under high-vacuum conditions.

### 2.16. Statistical Analyses

Descriptive data were reported as means ± standard deviations. An analysis of variance (ANOVA) was conducted using the general linear model to assess both the main effects and interactions among the various factors, with a significance level set at 5%. For statistical comparison of the mechanical properties, one-way ANOVA was performed, followed by a Tukey post hoc test. Additionally, Principal Component Analysis (PCA) was employed to further analyze the data. All statistical analyses were carried out using IBM SPSS V22 software.

## 3. Results

### 3.1. Film Thickness

The thickness of edible films and coatings is a crucial parameter, as it directly influences other physical properties of the film, including water vapor permeability and optical characteristics [25]. The thickness of pectin films with varying glycerol concentrations was measured, yielding values of 0.066 ± 0.000 mm, 0.086 ± 0.001 mm, 0.283 ± 0.000 mm, 0.325 ± 0.000 mm, and 0.453 ± 0.001 mm, respectively (Table 2). These results indicate a significant increase in film thickness with higher glycerol content, consistent with the findings of Zakaria et al. [26], reported in his study about the effect of glycerol on thermoplastic potato starch film, which indicated that film thickness increased with rising glycerol content, likely due to its interaction with the polymer matrix and its capacity to alter the network structure and water retention. Our results are in line with those reported by Ertürk & Ay. [27], who examined the effects of varying plasticizer concentrations on rye-based films. They noted that increasing the amount of plasticizer resulted in a corresponding increase in film thickness. This phenomenon can be attributed to the swelling ability of plasticizers, which absorb moisture. As the plasticizer content increases, so does the swelling, resulting in thicker films. Specifically, the thickness of the films formulated with glycerol ranged from 145 to 232 μm, indicating a significant impact of glycerol concentration on film properties.

### 3.2. Moisture Content

A lower moisture content in the film enhances its effectiveness in inhibiting the growth of microorganisms. However, it is essential to maintain a balance, as films with zero or very low moisture levels cannot be produced without negatively affecting quality characteristics such as color and texture. Excessively dry films may become brittle, leaving the food surface vulnerable [28]. The addition of glycerol significantly influenced the moisture content of the films (*p* < 0.05), resulting in moisture levels of 18% ± 0.06, 21% ± 0.09, 21.59% ± 0.10, 22% ± 0.11, and 25% ± 0.14 for varying glycerol concentrations (Table 2). Glycerol was incorporated as a plasticizer to improve the flexibility and moisture-related performance of the films. Hernando et al. [29] observed similar results in their study on the effect of glycerol in oil palm trunk starch bioplastics, which were modified with citric-acid epoxidized palm oil oligomers. Syafiq et al. [30] found that adding plasticizers led to an increase in the water content of hydrocolloid films. This finding is consistent with the explanation provided by Shafqat et al. [31], which demonstrated that glycerol, due to its hydrophilic nature, retains water within the film matrix. The study indicated that higher concentrations of glycerol facilitate the adsorption of water molecules, primarily because plasticizers like glycerol tend to form hydrogen bonds with water. Our results are in agreement with the thickness analyses; this moisture retention leads to an increase in film thickness, as observed, where the thickness of films containing glycerol varied significantly.

### 3.3. Solubility

The water resistance of packaging materials is essential for preserving the shelf life of food products. The incorporation of glycerol significantly enhances the solubility of films in water (*p* < 0.05), increasing from 13.83% to 32% as glycerol concentration rises. Specifically, the solubility values are shown in Table 2. Our findings are consistent with the study of Abedini et al. [32] about the effect of glycerol on alginate/quince seed gum. It was reported that films with higher glycerol concentrations resulted in an increase in the water solubility. This behavior may also result from the plasticizing effect of glycerol, which alters the polymer network’s affinity for water [33]. As a result, glycerol facilitates greater moisture absorption, thereby increasing their solubility, significantly altering the physical properties of the films. Supporting this, research by Santhosh & Sarkar. [34] indicated that the glycerol molecules’ disruption in the cellulose network might be the root cause for the increased solubility of the films.

### 3.4. Swelling Ratio

Glycerol has a notable impact on the swelling rate of films, significantly enhancing their capacity to absorb water. The incorporation of glycerol into film formulations increases the swelling ratio due to its hygroscopic properties, allowing for greater water uptake. The swelling ratios of the films with various concentrations of glycerol ranged from 17.81% to 76.67% (Table 2). The results showed that the interaction between glycerol and water molecules promotes a greater degree of swelling, potentially affecting the integrity and functionality of the packaging material.

Research by Sothornvit and Krochta [35] also supports these findings, indicating that the addition of glycerol improves the swelling characteristics of various biopolymer films. Their work showed that higher gxlycerol concentrations led to a marked increase in the swelling capacity of whey protein films, highlighting the role of plasticizers in modifying the physical properties of biopolymer matrices. Additionally, Zhang et al. [36] found that hydrophilic additives like glycerol enhance water absorption in polymer films, which is crucial for food packaging applications where moisture barrier properties are vital.

### 3.5. Water Vapor Permeability

This method allows for an accurate assessment of the influence of glycerol concentration on the barrier properties of pectin films. The water vapor permeability (WVP) values of the samples demonstrate a clear trend, with glycerol-plasticized films exhibiting significantly lower permeability compared to the control sample (PC). The PC film showed the highest WVP at 3.64 × 10^−10^ g m^−1^ s^−1^ Pa^−1^ ± 0.08, while the addition of glycerol led to a progressive reduction in WVP. Among the glycerol-plasticized samples, PGLY3 had the lowest WVP at 2.04 × 10^−11^ g m^−1^ s^−1^ Pa^−1^ ± 0.01, followed by PGLY2 (2.65 × 10^−11^ g m^−1^ s^−1^ Pa^−1^ ± 0.01) and PGLY1 (3.26 × 10^−11^ g m^−1^ s^−1^ Pa^−1^ ± 0.01). However, PGLY4 deviated from this trend, showing a slight increase in WVP to 1.13 × 10^−10^ g m^−1^ s^−1^ Pa^−1^ ± 0.05, though it remained lower than the control sample, indicating that the observed reductions in WVP are statistically significant (*p* < 0.05).

This suggests that the amount of glycerol significantly influences the barrier properties of the films, with an optimal range for minimizing WVP (Table 2). In a recent study, Agustin et al. [37] explained that increasing the concentration of glycerol generally led to a decrease in the water vapor permeability (WVP) value. This effect occurs because higher glycerol concentrations result in a greater number of free hydroxyl groups that preferentially bind to bacterial cellulose (BC) and sodium alginate, leaving fewer hydroxyl groups available to interact with water vapor. The strong hydrogen bonding interactions between BC, sodium alginate, and glycerol inhibit water binding within the biocomposite, thereby reducing the potential for water migration.

The increase in WVP on PGLY4 can be explained by the study of Tarique et al. [38] on arrowroot starch films, which showed that increasing glycerol from 15% to 45% resulted in a significant increase in WVP due to the strong interactions between biopolymers, which allow for effective performance even at lower glycerol concentrations, resulting in a dense and compact starch network. Consequently, this leads to lower water vapor permeability values. However, increasing the glycerol content up to 45% enhances the mobility and flexibility of the starch network chains, resulting in structural changes that create a looser network. As a result, the film matrices become thinner, ultimately improving the water vapor permeability values of the films.

Based on our findings, the optimal glycerol content for minimizing WVP in pectin films appears to be around 5%, where the balance between plasticization and structural integrity is best achieved, leading to the lowest WVP values observed in our study.

### 3.6. Contact Angle Measurements

The hydrophilicity of the film can be evaluated by measuring its water contact angle. A smaller contact angle signifies a more hydrophilic film [39]. The effect of glycerol on contact angle values of the pectin-based films is given in Table 3. The water contact angle for all film samples remained below 90°, indicating that they possess a hydrophilic surface. As the glycerol content increases, the contact angle of added glycerol films decreases. This indicates that incorporation of glycerol into the films increases their hydrophilic nature. Our results are consistent with the findings of Sreekumar et al. [40], in their study about starch thermoplastics, who explained the decrease in contact angle by stating that, when a drop of water is placed on the surface of starch, the hydroxyl (-OH) groups in the starch form hydrogen bonds with the water molecules, facilitating rapid spreading. Consequently, this interaction leads to a lower contact angle value. According to Fu et al. [41], the water-binding capacity of plasticizers significantly influences the hydrophilicity of films. Glycerol, in particular, is known for its high water-holding capacity. Consequently, an increased glycerol content leads to a reduction in the hydrophobicity of the films.

### 3.7. Fourier Transform Infrared Spectroscopy (FT-IR)

**Figure 3:**
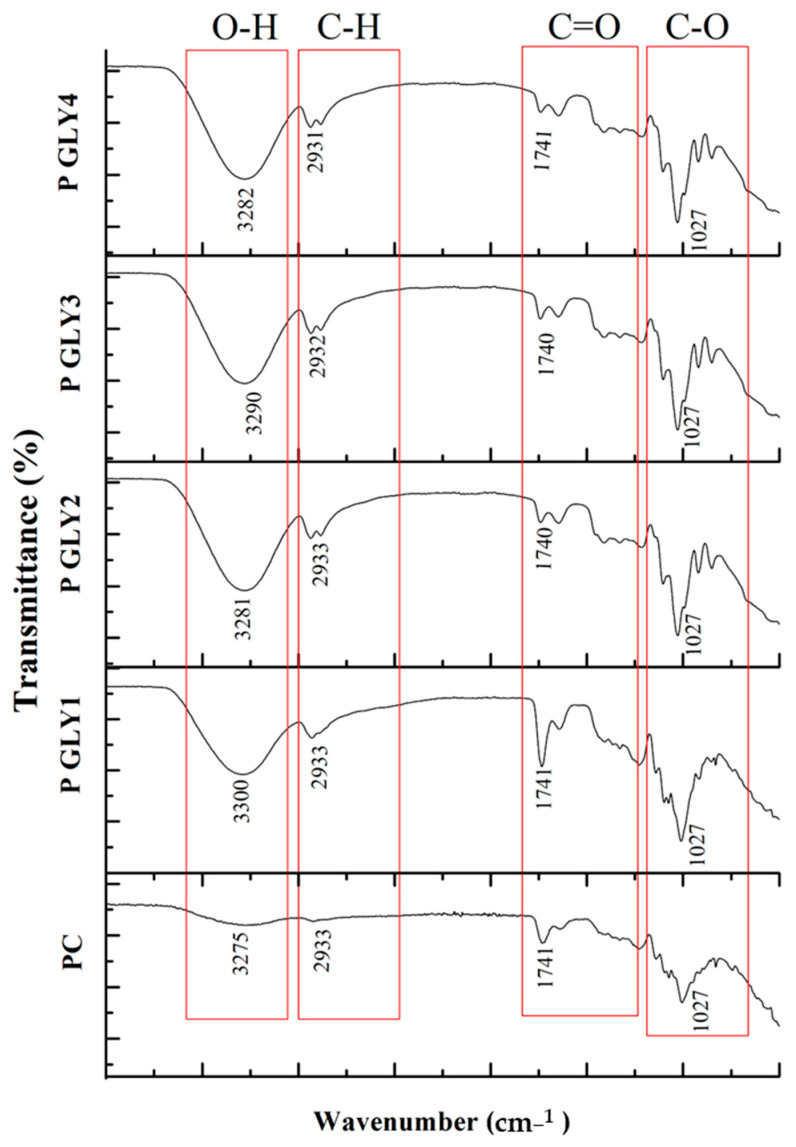
FT-IR spectrum of pectin films.

FTIR spectra of pectin-based films containing different concentrations of glycerol are provided in Figure 3. The broad absorption band at 3280 cm^−1^ is indicative of the O-H stretching vibration of hydroxyl groups, which are formed due to the hydrogen bonding. A peak at 2800–3000 cm^−1^ represents the stretching vibrations of C-H bonds in the hydrocarbon chains of the pectin and glycerol molecules. A peak at 1600–1800 cm^−1^ could be formed due to the carbonyl groups, which may be present in the pectin. The several peaks in the range of 1000–1200 cm^−1^ correspond to the stretching vibrations of C-O bonds, which are characteristic of polysaccharides like pectin. With the increase in the glycerol concentration in pectin films, the intensity of the O-H stretch increases due to the additional hydroxyl groups from glycerol. The position of the C-O stretch band shifts slightly due to interactions between the pectin and glycerol molecules. The observed peak at (~1740 cm^−1^) is attributed to the stretching vibration of the methyl esterification of the carbonyl group (C=O) of pectin [42]. New bands appear in the spectrum, indicating the formation of new chemical bonds or interactions between pectin and glycerol. According to Cao et al. [43], as the concentrations of glycerol increase, the O-H stretching bands become sharper and shift to lower wavenumbers. This phenomenon can be attributed to two main factors: first, the increase in glycerol concentration leads to a higher number of hydroxyl bonds, which sharpens the O-H peak. Second, the formation of hydrogen bonds between glycerol and polymer molecules causes the O-H stretching vibrations to shift to lower wavenumbers. This is in accordance with the previous analysis, which confirms the formation of intermolecular hydrogen bonds between pectin and glycerol, as shown by the absorption peak in the 3000–3700 cm^−1^ range. This interaction leads to a reduction in the water vapor permeability (WVP) value of the biocomposite. Comparable findings have also been observed in protein-based films made from rye combined with bacterial cellulose [44].

### 3.8. Optical Properties

Transparency refers to a film’s ability to exhibit clarity based on its capacity to transmit light, which is crucial when it is used as a surface coating or wrapping for food products [45]. The significant absorption observed in the UV range suggests that these films could serve as effective barriers against UV light when used in food packaging applications. The transparency values of the films ranged from 1.624 to 0.753 (*p* < 0.05; Table 3), with the PC and PGLY1 films demonstrating comparatively higher transparency than other samples, while the 30% glycerol film exhibited the lowest transparency. Pectin films are typically transparent; however, the addition of various additives has been reported to decrease their transparency. Furthermore, the opacity of the film was directly related to the film’s thickness [46]. An increment in the dry matter content of pectin films leads to a greater number of established bonds and an increase in film thickness. This, in turn, decreases light penetration and reduces transparency [47]. Also, in plasticized films, the presence of glycerol and sorbitol can disrupt the film’s structure, creating numerous pores of varying sizes on the surface. This structural alteration results in increased water permeability, which further reduces transparency [43] in accordance with the PGLY4 film. Our results indicate that an increasing glycerol concentration led to decreased opacity, suggesting enhanced transparency of the films. A similar trend was observed in a previous study by Mohammed et al. [48], which reported that, with the increasing content of plasticizer, the opacity of the film increased. These results were also in accordance with a previous study [25].

The transmittance results for the five pectin films with varying glycerol concentrations reveal a notable trend in light transmission (Figure 2). The film PC exhibited the highest peak transmittance at approximately 62.50% at 280 nm, followed closely by the PGLY1 film, which showed a peak of around 61.25%. In contrast, the films with higher glycerol concentrations demonstrated significantly lower transmittance values, with the PGLY2 film reaching a peak of 31.03% and the PGLY3 and PGLY4 films demonstrating the same transmittance value of 28.71 and 28.64%, respectively (Table 4). This pattern suggests that an increasing glycerol concentration correlates with reduced light transmittance, indicating an increase in opacity as glycerol levels rise.

### 3.9. Mechanical Properties

The mechanical properties of pectin-based films with varying glycerol concentrations were analyzed to determine the impact of glycerol as a plasticizer on film performance. The results of the five pectin-based films are summarized in Table 5. The tensile strength exhibited an inverse relationship with glycerol concentration (*p* < 0.05). The control sample had a tensile strength of 2.10 MPa, which increased to 20.43 MPa for PGLY1. However, as the glycerol concentration increased beyond this point, tensile strength decreased significantly, PGLY4 showing a low value of 0.87 MPa. This decline indicates that higher glycerol levels compromise the film’s ability to withstand stress. A high concentration of glycerol will weaken the strength of intermolecular forces, which enhances the mobility between molecular chains and results in an increase in elongation [49]. According to Geleta et al. [25], the high tensile strength observed at low glycerol concentrations may be attributed to the predominance of strong hydrogen bonds formed through polymer interactions, which outweigh the attractions between polymer and glycerol. Additionally, glycerol reduces the strong intramolecular attractions between polymer chains, facilitating the formation of hydrogen bonds between glycerol and polymer molecules. High variability in tensile strength measurements often stems from sample heterogeneity, which can arise from factors such as inconsistent film drying conditions, fluctuating temperatures, humidity, or curing times.

The elongation at break (EAB%) values of all glycrol-incorporated pectin films were found to be higher than those of the control film. The glycerol incorporated film at 5wt% (PGLY1) demonstrated significantly (*p* < 0.05) higher EAB compared to the other samples, indicating enhancement in mechanical properties. Liu et al. [50] also found that incorporating glycerol at concentrations of 5% (*w*/*w*) and above into starch–chitosan films led to increased EAB values. The elongation value of the PGLY1 sample was 20.62% while it was 1.25% for the control sample (PC). However, the elongation of films exhibited a significant reduction at higher glycerol concentrations (10–30 wt%) compared to the PGLY1 sample. The PGLY2, PGLY3, and PGLY4 showed values of 6.16%, 5.28%, and 2.90%, respectively. This result suggests that glycerol initially enhances the flexibility of the control film; however, excessive amounts may lead to a reduction in the structural integrity of the films. Our findings are in agreement with the research of Paudel et al. [13], who investigated the effect of glycerol and sorbitol on cellulose-based biodegradable films. They reported that the EAB for glycerol-plasticized cellulose films reached a maximum of 13.1%, significantly higher than the control films, which exhibited EAB values of only 5.1%.

The Young’s modulus values revealed a complex interaction with glycerol concentration, *p* < 0.05. The control sample had a modulus of 1.09 MPa, which increased to 2.83 MPa for PGLY1 but then peaked at 22.33 MPa for PGLY2 before dropping to 6.45 MPa for PGLY4. This behavior suggests that moderate glycerol content can enhance stiffness; however, excessive plasticizer use leads to a softer film structure.

The post hoc test is conducted to determine which specific pairwise comparisons among the means of the measured film properties contribute to the overall significant differences identified in the ANOVA results [51]. These results indicate significant differences in the mechanical properties of pectin-based films with varying glycerol concentrations. The Young’s modulus was found to be insignificant for the control film PC with the films PGLY1 and PGLY4, and also the film PGLY2 with PGLY3; these were significant for other pairwise comparisons. Strength and elongation between film PGLY1 and other film samples showed significant differences for all pairwise comparisons.

### 3.10. Principal Component Analysis for the Mechanical Properties

Principal Component Analysis (PCA) is one of the most widely used methods for multivariate data analysis. It is used to identify patterns in data and to present the data in a manner that emphasizes their similarities and differences. In high-dimensional datasets, where visual representation may be challenging, PCA serves as a powerful tool for data analysis, enabling the extraction of meaningful insights from complex information [52]. The resulting map (Figure 3) illustrates the correlations between films and their mechanical properties as determined by PCA.

**Figure 4:**
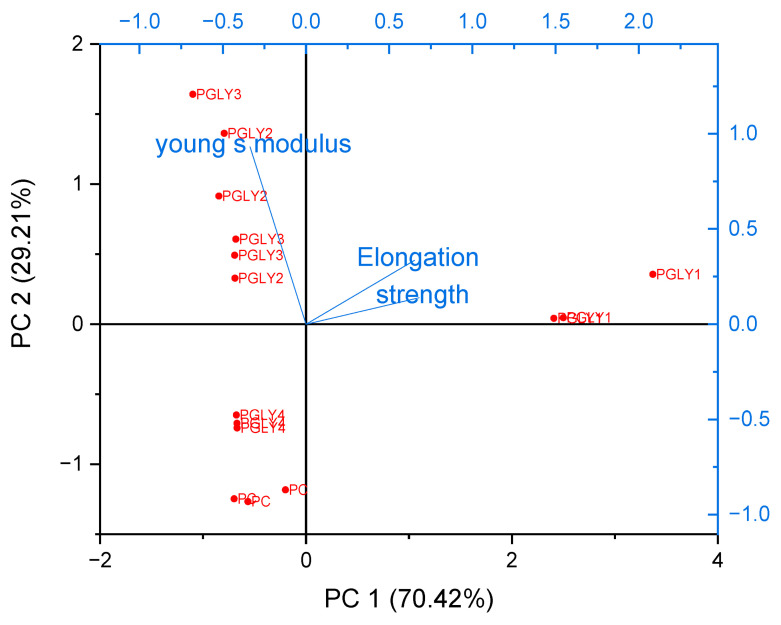
Principal Component Analysis (PCA) for the mechanical properties.

The results of PCA indicated that the quality of Figure 4 was good, with the axes PC1 and PC2 accounting for 70.42% and 29.21% of the variability, respectively, representing 99.63% of the information. The descriptor variables EAB and Young’s modulus form an angle, suggesting that they were independent of each other, similar to the relationship between strength and Young’s modulus. Conversely, elongation and strength were very close together, indicating a strong correlation between these two properties. Notably, the PGLY1 film was characterized by significantly higher elongation and strength compared to other formulations. Looking at the second principal component, a correlation was determined between the PGLY2 and PGLY3 films that exhibited similar characteristics, primarily in terms of Young’s modulus. Thus, the films PC and PGLY4 were close to each other in the lower right quadrant, which indicates a negative correlation. Based on the results obtained from PCA, it can be concluded that the film formulation containing 5 wt% glycerol exhibits the most favorable mechanical properties, demonstrating optimal elongation and strength characteristics. While glycerol plays a crucial role as a plasticizer in enhancing flexibility, it is evident that its concentration must be carefully controlled. The findings suggest that, while glycerol cannot be entirely eliminated from the formulation, maintaining it at a specific percentage, such as 5 wt%, is essential for achieving an effective balance between flexibility and structural integrity in biodegradable films.

### 3.11. Burst Strength

Burst strength is defined as the maximum strength of a material that enables it to withstand bursting when subjected to pressure [53]. The burst strength of the films is shown in Table 6. The PGLY1 showed the highest strength (4272.27 g and 3.61 mm). The further addition of glycerol (>5 wt%) resulted in a decrease in the strength values as a function of plasticizer content. The initial increase in burst strength with 5 wt% glycerol can be attributed to improved chain mobility, which allows for better stress distribution across the material. However, at higher glycerol levels, the plasticizing effect may dominate, resulting in a softer film that cannot maintain its strength under pressure. The result is comparable to the study of Cazón et al. [54], who investigated the development of biocomposites based on bacterial cellulose, glycerol, and poly (vinyl alcohol). It was reported that glycerol served as an internal lubricant, facilitating the movement of polymeric chains by minimizing friction between cellulose fibers. The concurrent addition of polyvinyl alcohol and glycerol to bacterial cellulose films led to a notable increase in elasticity, attributable to the combined reinforcing and plasticizing effects of both components. However, the plasticizing effect of glycerol resulted in a decrease in puncture resistance that was particularly pronounced at higher concentrations of polyvinyl alcohol and glycerol.

### 3.12. Film Morphology

**Figure 5:**
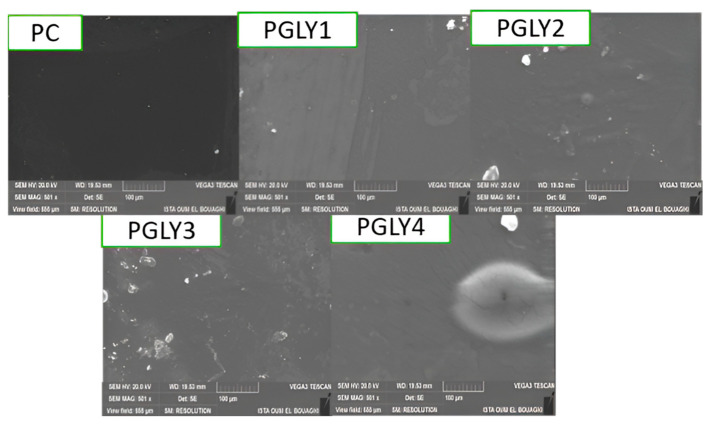
SEM micrographs of pectin film surfaces with varying glycerol content.

A scanning electron microscope (SEM) was used to examine the surface microstructures of pectin films with varying glycerol content (Figure 5). The SEM images revealed that pectin films without added glycerol exhibited smooth and uniform surfaces, free from cracks, breaks, or pores. However, as the glycerol content increased, the films displayed a less homogeneous morphology, with visible surface irregularities and impurities. These changes can be attributed to the plasticizing effect of glycerol, which disrupts the polymer matrix. Notably, the PGLY4 film, containing 30% glycerol, exhibited a pronounced halo, indicating significant phase separation or aggregation on the film surface due to the high plasticizer concentration.

## 4. Conclusions

This study demonstrated the significant effect of glycerol concentration on the various characteristics of pectin-based films enriched with lemon essential oil. The results highlighted glycerol’s crucial role as a plasticizer to develop pectin-based films with enhanced properties. The barrier, mechanical, optical, structural, and wettability properties of the films were influenced by the glycerol content. The water contact angle values showed that the surface hydrophilicity of pectin-based films increased with the incorporation of glycerol. The 5 wt% addition of glycerol enhanced the tensile strength of pectin film from 1.95 ± 2.49 MPa to 20.46 ± 4.61 MPa. However, tensile strength demonstrated an inverse relationship with further increase of glycerol concentration (10–30 wt%), underscoring the need for careful optimization. The optimal plasticizer content was determined as 5 wt% due to it providing a balance between mechanical strength and flexibility, as also supported by PCA analysis.

Notably, our findings demonstrate that optimal performance in terms of mechanical strength, solubility, and water vapor permeability can be achieved with a glycerol concentration as low as 5%, which is substantially lower than the 25–30% commonly reported in starch-, alginate-, and whey protein-based films. This suggests that the presence of lemon oil, combined with the intrinsic properties of pectin, may lead to synergistic effects that reduce the need for a high plasticizer content. Such behavior highlights the distinct structural and functional dynamics of our system compared to conventional biopolymer films. This study provides significant insights into the effects of glycerol concentration on the mechanical and barrier properties of pectin films. The findings highlight the potential of these films for applications in food active packaging and biomedical fields, where biodegradability and controlled moisture barrier properties are crucial. The optimal glycerol content of around 5% offers a promising balance between flexibility and barrier performance, making these films suitable for protecting food products from moisture while maintaining freshness and safety. However, this study has some limitations, including the lack of detailed analysis on the molecular interactions between glycerol, pectin, and lemon oil, as well as the absence of long-term stability and biodegradability assessments under real storage or environmental conditions. Future studies could explore the optimal films’ biodegradability in soil, water, or compost environments. In addition, the films’ interaction with suitable food products could be investigated to show the potential effect of the film to maintain quality and extend shelf life.

## Figures and Tables

**Table 1:** Composition of pectin-based films with varying glycerol concentrations *.

Sample Code	Pectin (% *w*/*v*)	Essential Oil (% *w*/*v*)	Glycerol (% wt of Polymer)	Citric Acid (% *w*/*w*)
PC	5	1	0	10
PGLY1	5	1	5	10
PGLY2	5	1	10	10
PGLY3	5	1	20	10
PGLY4	5	1	30	10

* The percentages reflect the formulation used to prepare each film sample.

**Table 2:** Properties of pectin-based films.

Film	Thickness (mm)	Moisture Content (%)	Solubility (%)	Swelling Ratio (%)	WVP (g m^−1^ s^−1^ Pa^−1^)
PC	0.066 ± 0.002	18.00 ± 0.06	13.83 ± 0.28	17.81 ± 0.50	3.64 × 10^−1^⁰ ± 0.08
PGLY1	0.086 ± 0.001	21.00 ± 0.09	19.79 ± 0.42	31.81 ± 0.61	3.26 × 10^−11^ ± 0.01
PGLY2	0.283 ± 0.005	21.59 ± 0.10	25.74 ± 0.54	48.96 ± 0.96	2.65 × 10^−11^ ± 0.01
PGLY3	0.325 ± 0.000	22.00 ± 0.11	26.48 ± 0.59	56.35 ± 1.12	2.04 × 10^−11^ ± 0.01
PGLY4	0.453 ± 0.001	25.00 ± 0.14	32.00 ± 0.67	76.67 ± 1.24	1.13 × 10^−1^⁰ ± 0.05

All values are means of triplicates ± standard deviation.

**Table 3:** Water contact angle of different pectin-based films.

Films	Contact Angle °	Image
PC	PC 69.19 ± 0.67	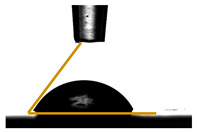
PGLY1	56.98 ± 0.69	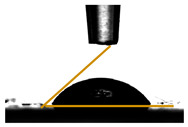
PGLY2	28.98 ± 0.30	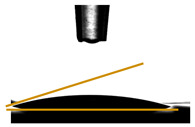
PGLY3	22.69 ± 0.70	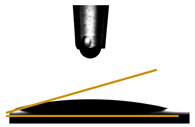
PGLY4	18.54 ± 0.74	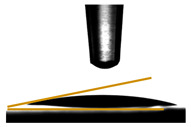

All values are means of triplicates ± standard deviation.

**Table 4:** Optical properties of pectin-based films.

Film Sample	Transmission (%)	Transparency UV Range (280 nm)	Transparency Visible Range (600 nm)
PC	62.50 ± 0.25	72.55 ± 0.50	1.624 ± 0.02
PGLY1	61.25 ± 0.30	78.30 ± 0.40	1.246 ± 0.03
PGLY2	31.03 ± 0.20	59.52 ± 0.60	0.814 ± 0.01
PGLY3	28.71 ± 0.15	45.06 ± 0.50	0.795 ± 0.02
PGLY4	28.64 ± 0.12	45.05 ± 0.45	0.753 ± 0.01

All values are means of triplicates ± standard deviation.

**Table 5:** The elongation, strength, and Young’s modulus of pectin films.

Film Samples	Elongation (%)	Strength (MPa)	Young’s Modulus (MPa)
PC	1.25 ± 0.93 ^a^	2.10 ± 1.56 ^a^	1.09 ± 0.40 ^a^
PGLY1	20.62 ± 2.19 ^d^	20.43 ± 3.89 ^b^	2.83 ± 0.48 ^a^
PGLY2	6.16 ± 1.13 ^bc^	2.66 ± 0.51 ^a^	22.33 ± 5.13 ^b^
PGLY3	5.28 ± 1.11 ^c^	2.40 ± 0.65 ^a^	21.75 ± 6.93 ^b^
PGLY4	2.90 ± 1.00 ^a^	0.87 ± 0.45 ^a^	6.45 ± 0.45 ^a^

All values are presented as means of quintuplicate measurements ± standard deviation. Statistical analysis was conducted using ANOVA, followed by Tukey’s post-hoc test for further comparison. Means in the same column with different superscript letters are significantly different (*p* < 0.05).

**Table 6:** Burst strength for pectin films.

Film Sample	Burst Strength (g)	Distance at Burst (mm)
PC	771.70 ± 2.31	0.930 ± 0.03
PGLY1	4272.27 ± 128.17	3.610 ± 0.11
PGLY2	673.25 ± 20.18	3.560 ± 0.11
PGLY3	559.40 ± 16.78	3.568 ± 0.11
PGLY4	219.20 ± 6.56	1.570 ± 0.05

All values are means of triplicates ± standard deviation.

## Data Availability

The original contributions presented in the study are included in the article, further inquiries can be directed to the corresponding author.
